# Serum inflammatory biomarkers are associated with increased choroidal thickness in keratoconus

**DOI:** 10.1038/s41598-023-37472-8

**Published:** 2023-07-05

**Authors:** João Pinheiro-Costa, Mário Lima Fontes, Carla Luís, Sandra Martins, Raquel Soares, Dulce Madeira, Fernando Falcão-Reis, Ângela Carneiro

**Affiliations:** 1 Department of Ophthalmology, Centro Hospitalar Universitário São João, Porto, Portugal; 2 Department of Biomedicine, Faculty of Medicine, University of Porto, Alameda Prof. Hernâni Monteiro, 4200-319 Porto, Portugal; 3 i3S - Institute of Research and Innovation in Health, University of Porto, Porto, Portugal; 4 Department of Clinical Pathology, Centro Hospitalar Universitário São João, Porto, Portugal; 5 EPIUnit - Institute of Public Health, University of Porto, Porto, Portugal; 6 Department of Surgery and Physiology, Faculty of Medicine, University of Porto, Porto, Portugal; 7 CINTESIS - Center for Health Technology and Services Research, Porto, Portugal

**Keywords:** Biomarkers, Medical research

## Abstract

Inflammation may play a significant role in Keratoconus (KC), but the relationship between inflammatory markers and choroidal thickness (CT) is unknown. The purpose of this study was to evaluate serum inflammatory markers and correlate them with the choroidal profile of KC patients and control subjects. Forty patients with KC and 26 age-matched control subjects were enrolled in a cross-sectional case–control study. Choroidal profile was studied with a Spectralis Heidelberg apparatus and venous blood samples were collected. Neutrophil/lymphocyte ratio (NLR), monocyte/HDL ratio (MHR), platelet/lymphocyte ratio (PLR) and systemic immune inflammation index (SII) were calculated. Serum inflammatory biomarkers IL-1, IL-6 and TNF-alfa were also analyzed. KC group presented thicker choroids in each evaluated point when compared to the control group (subfoveal CT 417.38 ± 79.79 vs 299.61 ± 76.13, *p* < 0.001 for all measured locations). Mean values of NLR, PLR and SII were significantly higher in patients with KC (NLR *p* = 0.001; PLR *p* = 0.042; SII *p* = 0.007). Although KC patients presented higher mean levels of MHR, IL-1, IL-6 and TNF-α than control group, no significant differences were achieved. Positive correlations were found between subfoveal CT and NLR and SII (0.408, *p* = 0.001 and 0.288, *p* = 0.019 respectively). The results presented are in favor of a relationship between the increased CT and inflammatory mechanisms in KC patients. The elevated serum inflammatory indices NLR, SII and PLR provide additional evidence of a role for systemic inflammation in the pathophysiology of KC.

## Introduction

Keratoconus (KC) is a cornea disease that is characterized by progressive thinning and cone shaped protrusion. It usually shows bilateral involvement and manifests at puberty or early adulthood^1–5^.

Despite being traditionally regarded as a non-inflammatory condition, recent research has revealed significant concentrations of local and systemic proinflammatory mediators in KC patients, indicating a possible role for inflammation^6–8^. The tear film and corneal epithelium of these patients was found to contain traces of cytokines IL-1, IL-6 and TNF-a and protease MMP-9, responsible for stromal thinning and extracellular matrix breakdown^9–11^ which may be crucial in explaining KC pathophysiology^6–8,12^.

In addition to local activation of inflammatory pathways, there is growing evidence that systemic inflammatory markers and systemic oxidative stress are elevated in patients with KC^6,13,14^. High levels of Neutrophil/lymphocyte ratio (NLR), monocyte/high-density lipoprotein cholesterol ratio (MHR), platelet/lymphocyte ratio (PLR) and systemic immune-inflammation index (SII), recognized indicators of systemic inflammation, were consistently found in recent studies^14–18^. Furthermore, KC patients also showed higher serum levels of several proinflammatory markers (IL-1B, IL-6, TNF-α, MMP-9 and NF-kB), higher serum levels of total oxidant status and oxidative stress index, and reduced serum levels of Vitamin D^13,19,20^. An overexpression of Toll-like receptor 2 (TLR2) in blood monocytes and neutrophils, as well as in corneal and conjunctival epithelial cells of KC patients, was recently demonstrated^19,21,22^. The systemic innate immune TLR2 overexpression was correlated with the increase in inflammatory mediators and NF-kB factors in serum^19^. Moreover, the patients with KC have shown a lower concentration of Lactoferrin (LTF) in tear fluid than control subjects, and this tear LTF reduction evidencing a strong correlation with TLR2 overexpression at systemic and ocular surface level^23^. This dysregulation of LTF and TLR2 in the ocular surface of KC patients seems to contribute to KC severity by maintaining a detrimental chronic immune—inflammatory state^23^.

Our group's previous research, demonstrated the presence of an increase in choroidal thickness (CT) in KC patients^24,25^. Although the precise mechanisms behind the increase in their CT profile are not certain, we believe that inflammatory mechanisms may play a role^24^.

Moreover, Gutierrez–Bonet et al.^26^ recently stated that the increase in CT of KC patients occurred predominantly due to an increase in vascular area over the stromal area (increase in the corrected choroidal vascularity index of 7.19% in KC). These results seem to indicate vascular dilation as the major component of choroidal thickening.

Therefore, the purpose of this study was to evaluate serum inflammatory biomarkers and their correlation with the choroidal profile of KC patients and control subjects.

## Results

We prospectively studied 40 patients with KC (14 female; mean age 23.25 ± 4.23 years) and 26 control subjects (11 female; mean age 22.96 ± 3.64 years).

No age- and sex-related statistical differences were detected between patients with KC and control subjects. However, patients with KC showed more frequent history of Atopy and eye rubbing habits (Table 1). Twenty-nine KC patients (72.5%) and 7 control subjects (26.9%) reported atopic disease (allergic rhinitis, atopic dermatitis or asthma) (*p* < 0.001).

**Table 1:** Clinical characteristics, topographic variables, choroidal thickness and laboratory parameters analyzed in patients with keratoconus and control subjects.

	Keratoconus (n = 40)	Control (n = 26)	*p*-value*
Population characteristics			
Age (years)	23.25 (4.23)	22.96 (3.64)	0.769
Sex, female (%)	35.0 (n = 14)	42.3 (n = 11)	0.550
Right Eye (%)	72.5 (n = 29)	84.6 (n = 22)	0.251
Atopy (%)	72.5 (n = 29)	26.9 (n = 7)	**< 0.001**
Eye rubbing habits	2.48 (0.72)	0.38 (0.64)	**< 0.001**
SphEq (D)	− 2.18 (2.11)	− 1.58 (1.77)	0.214
KC Classification	2.41 (1.07)	0.00 (0.00)	**< 0.001**
AL (mm)	23.85 (0.84)	24.23 (0.93)	0.093
AL adjusted (mm)	20.07 (0.74)	20.59 (0.77)	**0.009**
Kmax (D)	58.31 (11.91)	44.02 (1.36)	**< 0.001**
PachyMin (μm)	444.58 (48.97)	539.32 (32.20)	**< 0.001**
D Index	9.99 (6.23)	0.94 (0.45)	**< 0.001**
Choroidal thickness			
N1500 (μm)	349.80 (82.69)	255.96 (70.90)	**< 0.001**
N1000 (μm)	382.98 (81.61)	280.42 (72.75)	**< 0.001**
N500 (μm)	404.45 (80.64)	294.19 (74.70)	**< 0.001**
Subfoveal (μm)	417.38 (79.79)	299.61 (76.13)	**< 0.001**
T500 (μm)	418.98 (79.27)	295.58 (74.97)	**< 0.001**
T1000 (μm)	415.28 (79.80)	290.69 (73.07)	**< 0.001**
T1500 (μm)	406.43 (80.91)	288.50 (72.28)	**< 0.001**
Laboratory parameters			
WBC (× 10^6^/mL)	6.67 (1.59)	6.45 (1.37)	0.549
Neutrophil count (× 10^6^/mL)	3.88 (1.17)	3.32 (1.10)	0.055
Lynphocyte count (× 10^6^/mL)	2.03 (0.54)	2.40 (0.61)	**0.016**
Monocyte count (× 10^6^/mL)	0.50 (0.18)	0.49 (0.14)	0.749
Platelet count (× 10^6^/mL)	236.50 (47.36)	240.85 (39.50)	0.688
HDL-C (mg/dL)	52.38 (10.05)	62.85 (12.10)	**0.001**
CRP (mg/L)	3.01 (4.43)	2.58 (2.35)	0.373
IL-1 (pg/mL)	6.59 (9.12)	5.79 (9.55)	0.748
IL-6 (pg/mL)	1.62 (1.64)	1.16 (1.08)	0.190
TNF-alpha (pg/mL)	46.28 (34.39)	37.68 (27.14)	0.282
NLR^**†**^	1.99 (0.64)	1.45 (0.55)	**0.001**
MHR (× 10^2^)^**†**^	0.98 (0.39)	0.81 (0.30)	0.050
PLR (/10^2^)^**†**^	1.22 (0.31)	1.06 (0.30)	**0.042**
SII (/10^2^)^**†**^	4.70 (1.80)	3.53 (1.59)	**0.007**

Results are expressed as mean ± SD for continuous variables. Female gender, atopy and right eyes are expressed as count and percentage.

Choroidal Thickness measurements undertaken at subfoveal choroid (Subfoveal), nasal 500 μm (N500), 1000 μm (N1000) and 1500 μm (N1500) and temporal 500 μm (T500), 1000 μm (T1000), 1500 μm (T1500).

*SphEq* spherical equivalent, *KC* keratoconus, *AL* axial length, *Kmax* maximum keratometry, *PachyMin* minimum pachymetry; *D-Index* Belin/Ambrósio deviation index, *D* diopter.

*IL* interleukin, *TNF-alfa* tumor necrosis factor alpha, *WBC* white blood cells, *NLR* neutrophil to lymphocyte ratio, *MHR* monocyte to HDL-C ratio, *PLR* platelet to lymphocyte ratio, *SII* systemic immune inflammation index, *CRP* C-reactive protein.

*p < 0.05 was considered statistically significant.

^**†**^Cellular indices were all presented in the same order of magnitude for comparison reasons.

Significant values are in bold.

Thirty-seven KC patients (92.5%) reported moderate to severe eye rubbing habits (grade 2 or 3), in contrary 18 control patients (69.2%) reported no eye rubbing habits (grade 0) (*p* < 0.001).

Regarding KC classification using Pentacam HR Topographical Keratoconus Classification, 14 eyes (35.0%) presented mild or moderate disease (KC classification grade 1 or 2) and 26 eyes (65.0%) showed more severe disease (KC classification grade 3 or 4). Tomographic parameters of both groups are presented in Table 1.

Spherical equivalent and axial length (AL) results were comparable between groups; although when AL adjusted for the ACD was compared, KC eyes presented a shorter axial length (20.07 ± 0.74 vs 20.59 ± 0.77, *p* = 0.009).

### Choroidal thickness evaluation

Regarding CT, KC group presented thicker choroids in each evaluated point, when compared to the control group (*p* < 0.001 for all measured locations). In the subfoveal region CT was 417.38 ± 79.79 in KC group vs 299.61 ± 76.13 in control group. The results of CT of both groups are presented in Table 1.

**Figure 1:**
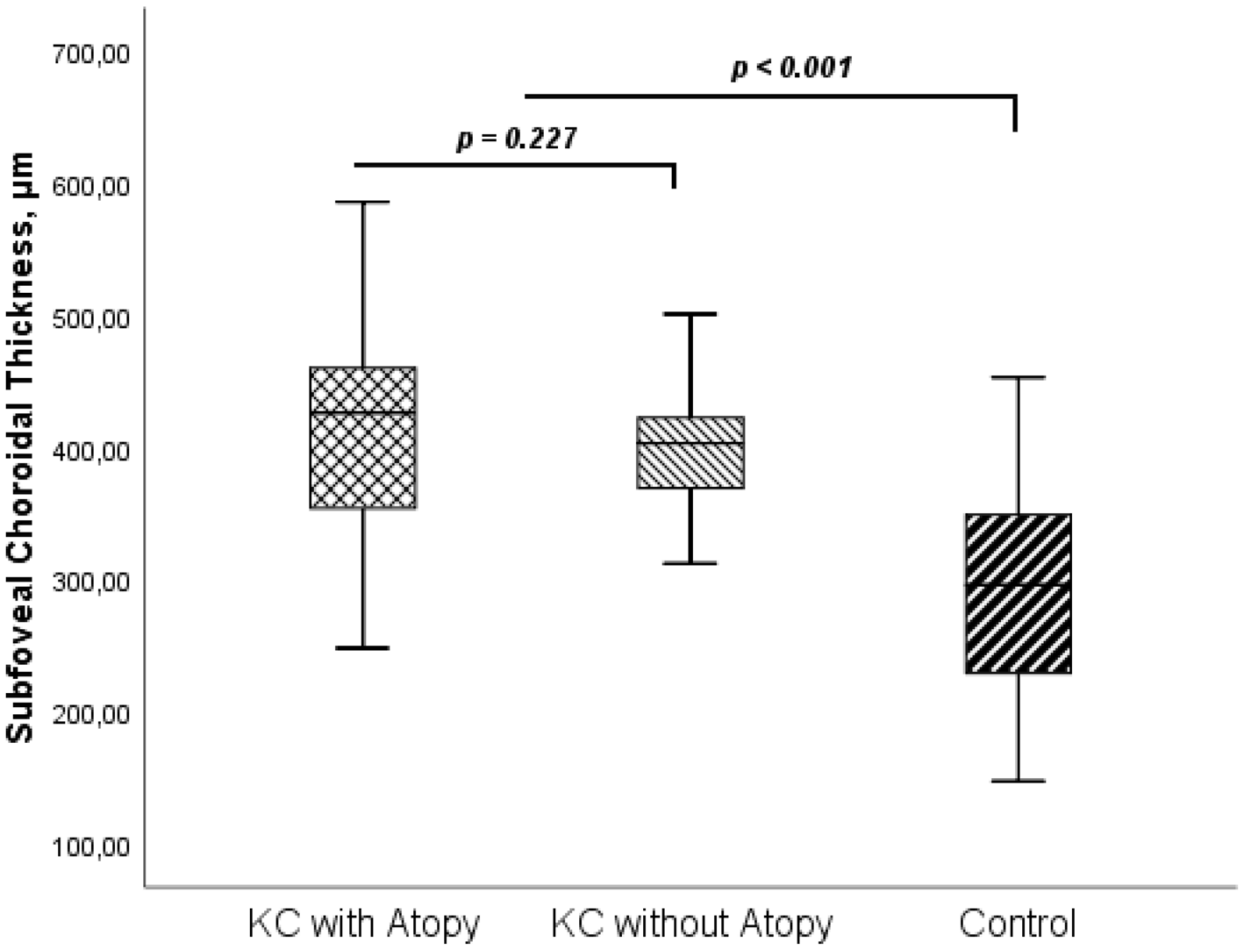
Subfoveal Choroidal Thickness in Keratoconus with and without Atopy and in control group. *p*-values are presented for comparison between groups (KC with Atopy vs KC without Atopy and KC vs Control). KC, Keratoconus.

Regarding the differences in atopic disease between groups, CT was also evaluated in KC with Atopy and without Atopy. No CT differences were found between KC patients with and without atopic disease (*p* > 0.05 for all measured locations). Subfoveal measurements are represented in Fig. 1 (425.17 ± 87.13 vs 396.82 ± 54.14, *p* = 0.227).

### Serum inflammatory indices (NLR, PLR, SII and MHR) and inflammatory molecules

**Figure 2:**
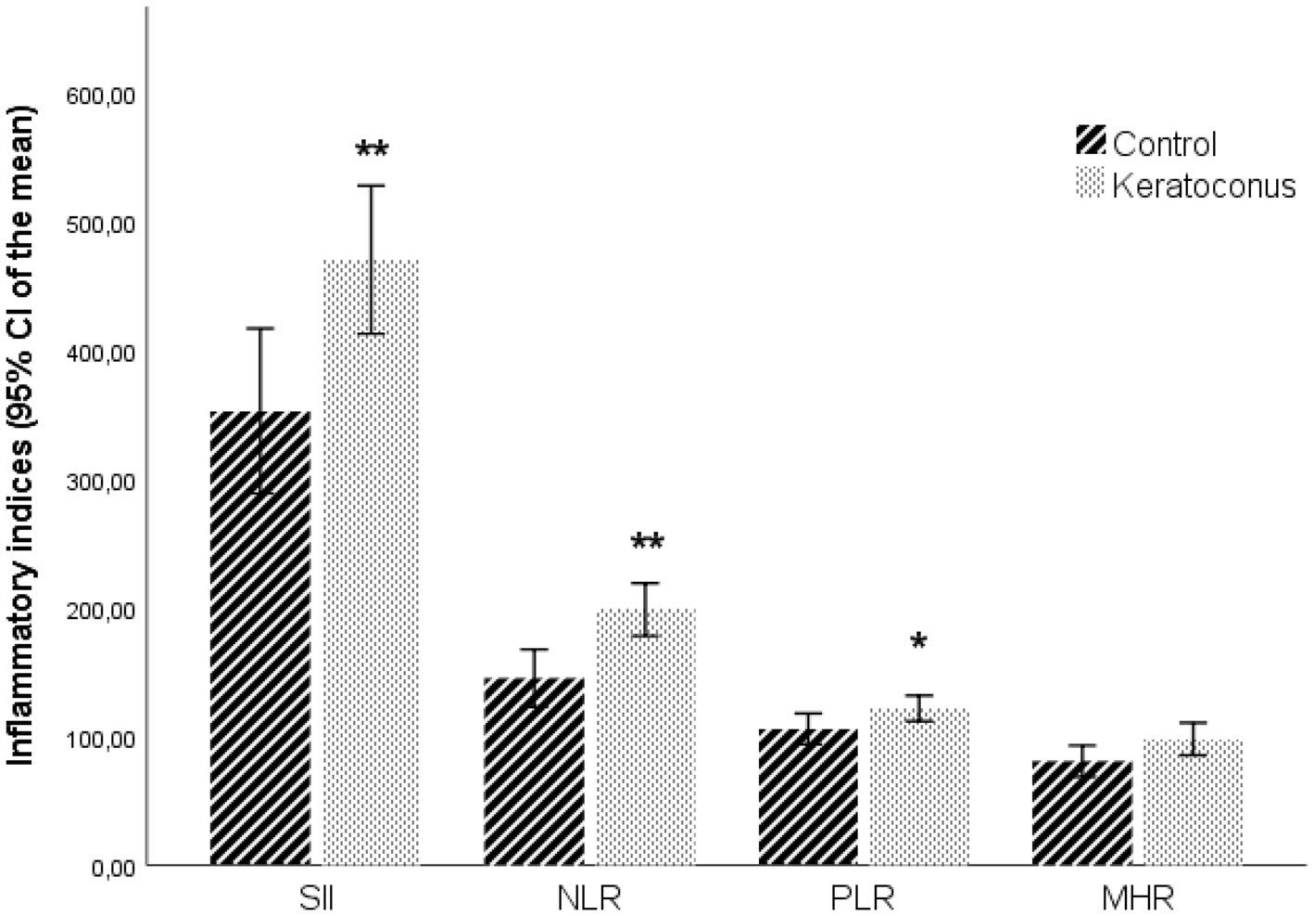
Inflammatory indices by study groups. Values represent the 95% CI for mean. **p < 0.01; * *p* < 0.05; MHR *p* = 0.05 compared to controls. For graphic proposals inflammatory indices were presented × 10^2^. NLR, Neutrophil to Lymphocyte Ratio; MHR, Monocyte to HDL-C ratio; PLR, Platelet to Lymphocyte Ratio; SII, Systemic Immune Inflammation Index.

Figure 2 shows that the mean values of NLR, PLR and SII were significantly higher in patients with KC, compared to control subjects (NLR *p* = 0.001; PLR *p* = 0.042; SII *p* = 0.007) (Table 1). MHR difference between groups did not reach a statistically significant value (*p* = 0.05).

**Figure 3:**
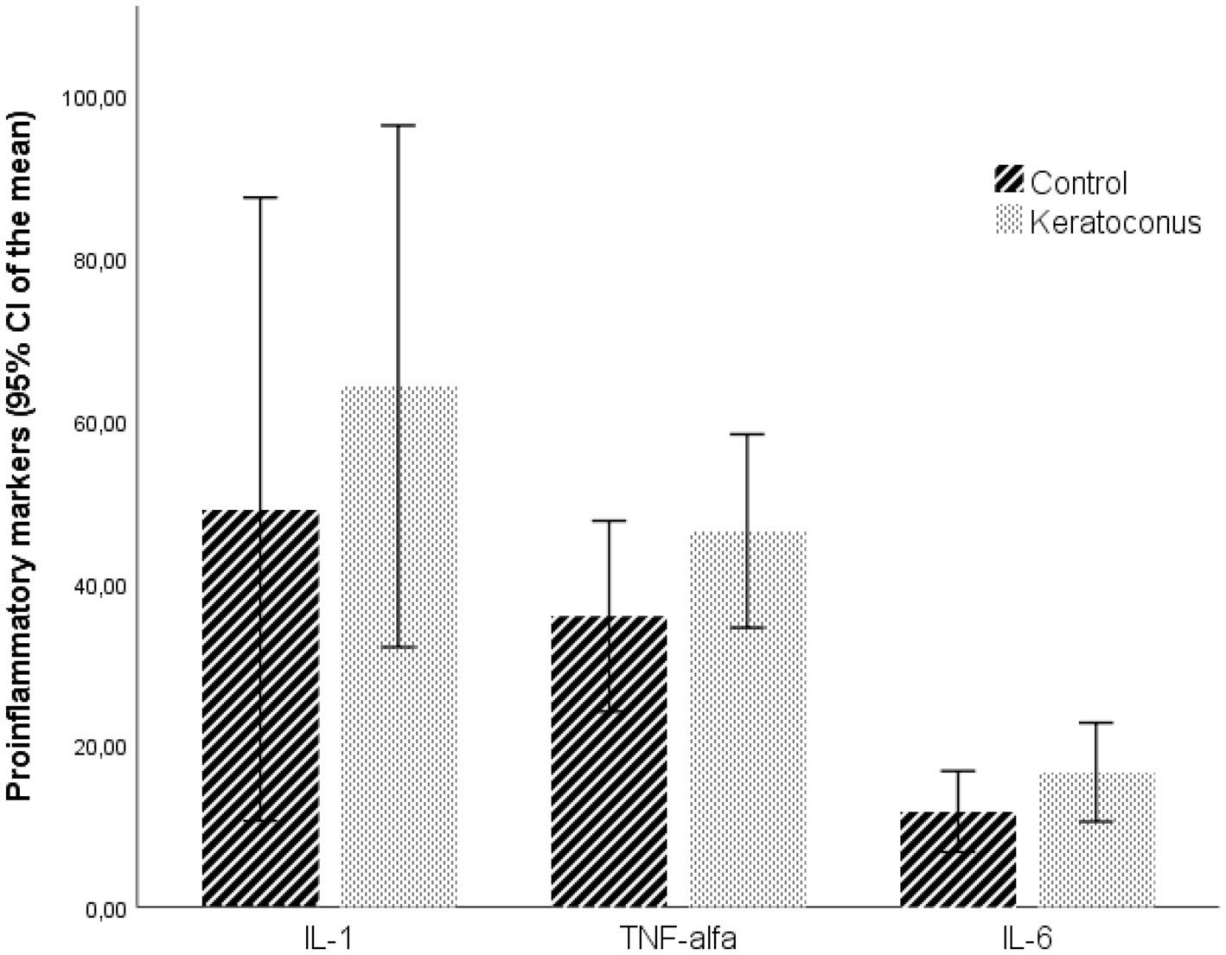
Proinflammatory markers by study groups. Values represent the 95% CI for mean. Values are tendentially higher in keratoconus group, although without statistical significance. IL-1 *p* = 0.748; TNF-alfa *p* = 0.282; IL-6 *p* = 0.190 compared to controls. For graphic proposals IL-1 and IL-6 values were presented × 10. IL, Interleukin; TNF-alfa, Tumor Necrosis Factor alpha.

Although KC patients presented higher mean serum levels of IL-1, IL-6 and TNF-α than the ones of the control group, the differences between groups were not significant (Fig. 3 and Table 1).

**Figure 4:**
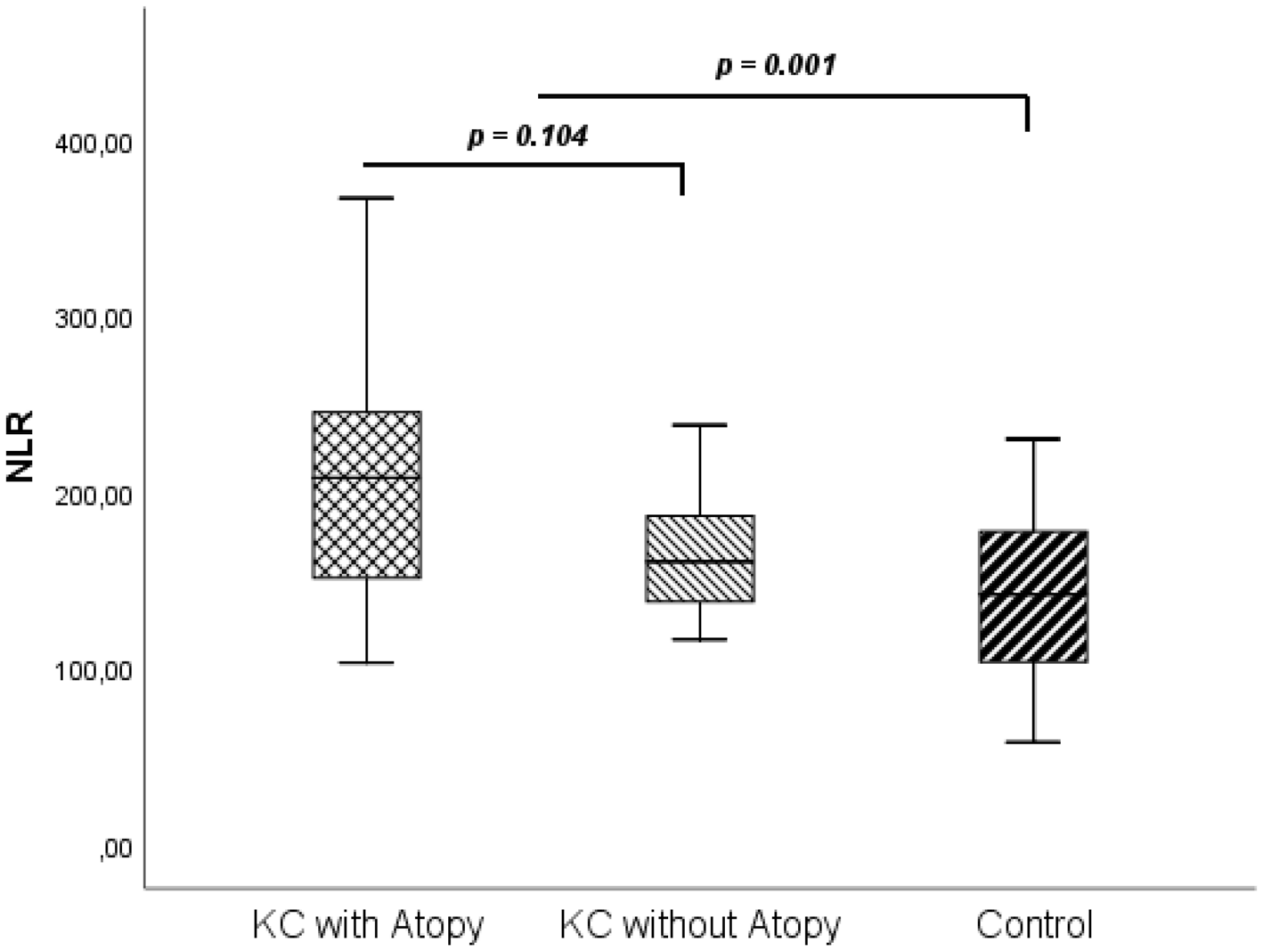
NLR in Keratoconus with and without Atopy and in control group. *p*-values are presented for comparison between groups (KC with Atopy vs KC without Atopy and KC vs Control). NLR, Neutrophil to Lymphocyte Ratio; KC, Keratoconus.

Once again, regarding the differences in atopic disease between groups, NLR, PLR and SII were also compared in KC with Atopy and without Atopy, and no differences were found between KC patients with and without atopic disease (*p* > 0.05 for the evaluated indices). For representative proposals NLR results are presented in Fig. 4 (2.08 ± 0.66 vs 1.74 ± 0.54, *p* = 0.104).

### Correlation between choroidal thickness and serum inflammatory indices (NLR, PLR, SII and MHR)

**Figure 5:**
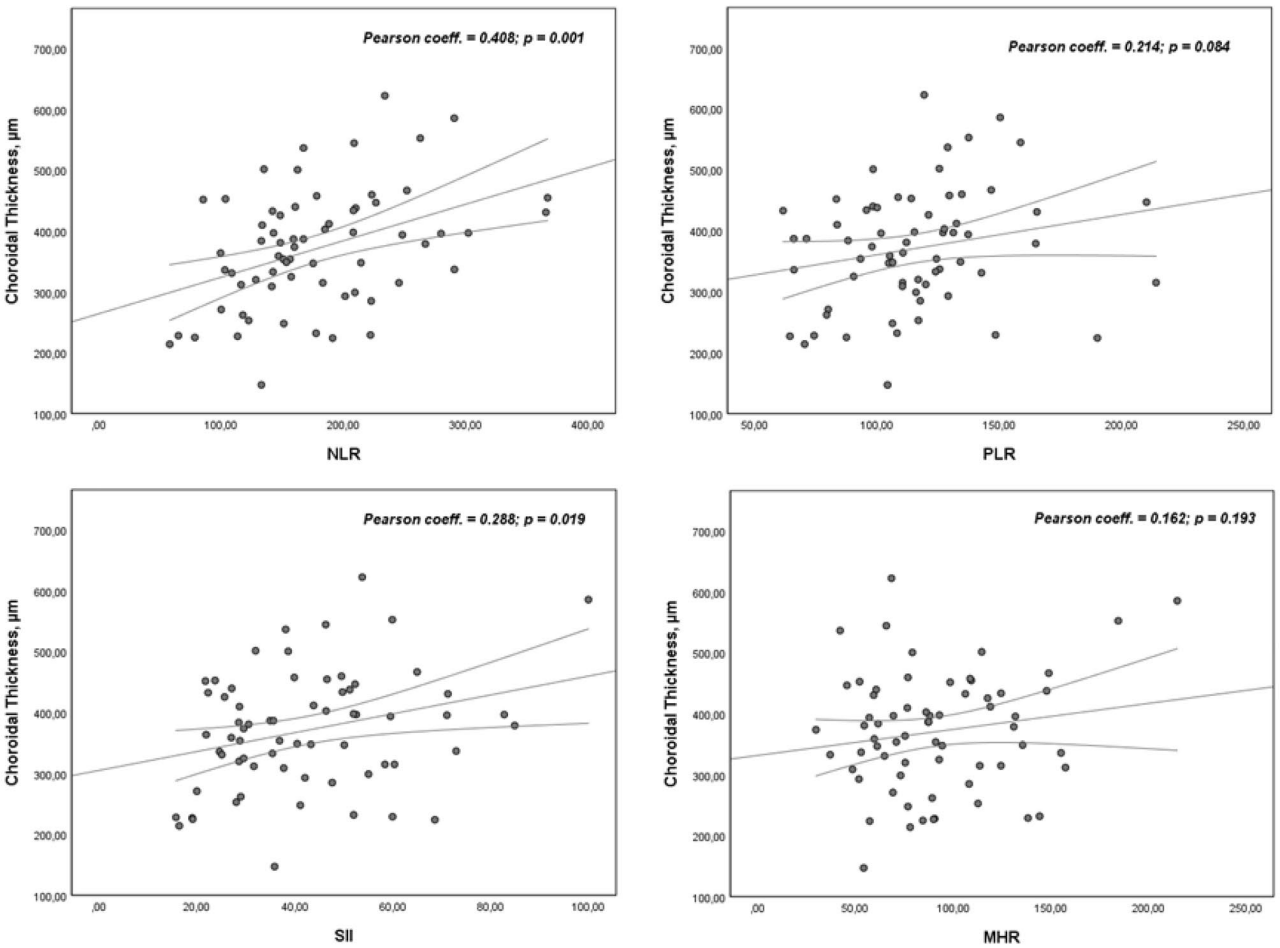
Correlations between Subfoveal CT with the levels of NLR, PLR, SII and MHR. Pearson coefficients and *p*-values are presented for each correlation. NLR, Neutrophil to Lymphocyte Ratio; MHR, Monocyte to HDL-C ratio; PLR, Platelet to Lymphocyte Ratio; SII, Systemic Immune Inflammation Index.

Figure 5 shows the correlations between Subfoveal CT with the levels of NLR, PLR, SII and MHR. We found a positive correlation between CT and NLR (0.408, *p* = 0.001) and a weak positive correlation between CT and SII (0.288, *p* = 0.019). No association was found between CT and both PLR and MHR indices.

Regarding the differences in atopic disease between groups, we performed a multiple linear regression analysis, adjusted by atopic disease, in order to analyze the independent influence of NLR and SII in Subfoveal CT. As shown in Table 2, no effects were observed for atopic disease on the influence of NLR and SII in Subfoveal CT.

**Table 2:** B estimate of Subfoveal Choroidal Thickness variation for a unit of NLR and SII in adjusted and unadjusted model for Atopy.

	B (CI 95%), *p*-value Unadjusted model for Atopy	B (CI 95%), *p*-value Adjusted model for Atopy
NLR	29.80 (− 2.15 to 61.76), 0.067	29.78 (− 2.63 to 62.20), 0.071
SII	5.65 (− 5.87 to 17.17), 0.331	5.59 (− 6.07 to 17.25), 0.341

Note that due to the collinearity between the different indices, the influence of each of them on Choroidal Thickness was assessed in a separate multiple linear regression model.

*NLR* neutrophil to lymphocyte ratio, *SII* systemic immune inflammation index.

Different correlations of CT and the other studied variables were tested during the analysis, and an interesting result, in accordance with our group previous publication^27^, was the good correlation between CT and eye rubbing (spearman´s Rho 0.507, *p* < 0.001).

### Correlation between choroidal thickness, NLR and SII and severity of KC

No association was found between the increase in CT, NLR and SII with the severity of KC (evaluated with the Pentacam HR Topographical Keratoconus Classification).

The Spearman’s Rho correlations coefficients were the following: Subfoveal CT 0.311, *p* = 0.051; NLR 0.101, *p* = 0.536; SII 0.057, *p* = 0.726. Although the increase of CT in KC group showed a tendency of correlation with the severity of the disease, the correlation did not reach a significant level.

### Multivariate analysis model for choroidal thickness variation

For descriptive purposes, a multivariate analysis was performed in order to demonstrate the influence of the various studied parameters on CT. The model included age and adjusted AL (the well-known influencers of CT), KC history, Atopy history, eye rubbing habits and NLR (the inflammatory marker with strong correlation with CT in our sample). The results are presented in Table 3. Adjusted R^2^ for the model was 34.3%.

**Table 3:** Multivariate linear regression analysis with an adjusted model for age, axial length adjusted, Keratoconus history, Atopy history, eye rubbing habits and Neutrophil to Lymphocyte Ratio.

	Β coefficient	95% Confidence Interval for B	*p*-value
Intercept	406.73	–	–
Age	1.72	− 3.30 to 6.75	0.496
AL adjusted	− 9.18	− 35.69 to 17.33	0.491
KC history	113.65	39.97 to 187.34	0.003
Atopy history	4.96	− 41.84 to 51.77	0.833
Eye rubbing habits	− 9.45	− 39.95 to 21.05	0.538
NLR	30.69	− 2.04 to 63.41	0.066

Dependent variable: Subfoveal Choroidal Thickness, μm. Adjusted R2 for the model was 34.30%

*KC* keratoconus, *AL* axial length, *NLR* neutrophil to lymphocyte ratio.

As shown in the model, KC history has a significant influence on the CT of the studied population, meanwhile Atopy history and eye rubbing habits seem not be determinant.

## Discussion

Recent publications on KC that suggest a role for inflammation in the disease pathophysiology and the newly demonstrated presence of an increased CT profile in KC patients have prompted questions regarding the link between inflammation and choroidal thickening^24–26^. Our group decided to evaluate serum inflammatory biomarkers and correlate them with the choroidal profile of KC patients.

In fact, a large number of studies provided evidence of increased levels of pro-inflammatory cells, cytokines and other inflammatory mediators in the tears of KC patients, whereas inflammatory suppressants seem to be reduced^6–10,12,23,28,29^. Mounting evidence suggests that the corneal milieu in KC may be impacted not only by the local activation of inflammatory pathways but also by systemic inflammation and systemic oxidative stress^7,13,19^. The pro-inflammatory profile may explain the finding of increased CT in KC eyes in contrast to a healthy population^24,25,30^.

Systemic inflammation monitored via the neutrophil-to-lymphocyte ratio was associated with progressive KC by Karaca et al.^14^. The authors argued that the increased frequency of neutrophils indicates pro-inflammatory conditions, and higher levels of neutrophils are associated with activation of proteolytic enzymes and MMPs, which may contribute to KC progression. More recently, other nonspecific systemic inflammatory markers were linked with KC. Elbeyli et al. demonstrated that SII, NLR, RDW, and PLR levels were significantly increased in patients with KC; furthermore, the results of Oltulu et al. showed that MHR and NLR values were significantly higher in the KC patients^17,18^.

As per the literature, in the present study, we found significantly higher values of NLR, SII and PLR in KC. These parameters, even nonspecific markers for diseases, can shed light on the role of systemic inflammation in the pathophysiology of KC.

The exact relation between these raised cellular indices and KC is unknown but may involve the presence of oxidative stress, chronic inflammation and endothelial dysfunction^15,17,18^. SII is one of the newly prognostic biomarkers based on platelets, neutrophils, and lymphocytes. Lymphocytes are known to play a crucial role inhibiting cell proliferation and migration. Lymphopenia indicates the ineffectiveness of the immune surveillance systems. High SII, consisting of high neutrophil and platelet as well as low lymphocyte counts, indicates inflammation activity that may be associated with KC^18^.

Beyond the inflammatory cellular indices, we evaluated serum levels of proinflammatory molecules that have been associated with KC in other studies^9,11,19^. Sobrino et al. demonstrated that patients with KC had higher serum levels of IL-1B, IL-6, TNF-α, MMP-9 and NF-κB. In addition, they found a strong correlation between TLR2 and TLR4 expression in both monocytes and neutrophils with serum levels of those proinflammatory molecules, claiming that such inflammatory response could be a consequence of the TLRs activation in KC patients^19^.

In the present study, although KC patients presented higher mean serum levels of IL-1, IL-6 and TNF-α, the differences between groups were not significant, and it was not possible to use those results to correlate with choroidal profile. The results of those proinflammatory molecules showed a great range of variability which makes impossible to reach significant differences with our sample size.

As expected, the choroidal profile results of the studied population are in accordance with previous published results^24–26,30–33^, demonstrating a thicker macular choroid in KC eyes compared to healthy age-matched controls (subfoveal CT 417.38 in KC group vs 299.61 in control group, *p* < 0.001 for all measured locations).

In the correlation analysis between subfoveal CT and serum inflammatory indices, positive correlations were found with NLR and SII. Although only moderately positive, the correlations are significative and are in favor of a relationship between the increased CT and inflammatory mechanisms.

In a recent publication, Gutierrez–Bonet et al.^26^ studied the increased CT in KC population with the use of choroidal binarization techniques. They demonstrated that both stromal and vascular area were thicker in KC, but there was a larger increase in vascular area, when compared to the stromal area in KC vs healthy controls, which involves an increase in the corrected choroidal vascularity index of 7.19%, when compared with healthy controls. The authors argued that these results may point toward vascular dilation and inflammatory stromal infiltration in KC patients contributing to the increase in CT, with vascular dilation being the major component of choroidal thickening^26^.

Moreover, Aydemir et al.^34^ not only observed an increased CT, but also found that the central retinal vascular caliber (arteries and veins), analyzed in the peripapillary region, were significantly increased in KC patients when compared to astigmatic and control groups. This finding could be another vascular effect of inflammation in KC patients, and help to support theories based on the inflammation affecting choroidal and retinal vascular parameters^34^.

Histological studies about some inflammatory diseases, such as Vogt–Koyanagi–Harada, have described inflammatory cells at stromal level with an increase in CT during inflammatory phase, which returns to normality after corticosteroid treatment^35–37^. These inflammatory cells activate the production of proinflammatory cytokines, the same that can be found elevated in blood or tear film of KC patients. These cytokines could be responsible for the activation of endothelial nitric oxide synthase, producing choroidal vascular dilation^26,38,39^.

If serum inflammatory biomarkers are associated with increased CT, and vascular dilation seems to be the major component of choroidal thickening, consequently, it is tempting to postulate that such increased CT could be a consequence of an inflammatory imbalance in those subjects^24–26^.

Aydemir et al. described that the CT and central retinal vascular caliber were progressively increased as the stage of KC advanced. But, in the present study, like in the results presented by Gutierrez–Bonet et al.^26^ although the increase of CT showed a tendency of correlation with the severity of the disease, that correlation did not reach a significant level.

Regarding the differences in atopic disease between groups, CT were also evaluated in patients with Atopy and without Atopy, but atopic disease seems not be determinant in the results of this particular sample. On this subject, previous studies by our group have demonstrated a tendency for a thicker choroid in atopic KC patients, when compared to non-atopic, suggesting a possible role for atopy in the choroidal profile of KC^27^. The small size of the study’s sample might help justify the absence of differences between groups.

The influence of eye rubbing on CT was tested and evaluated in several models. Although the grade of eye rubbing presented a good correlation with de CT and may influence it^27^, when eye rubbing is analyzed in conjunction with the presence/absence of KC and inflammatory indices (NLR or SII) it is completely masked (B coefficient -9.45, 95% CI for B -39.95 to 21.05, *p*-value 0.538). The effect of eye rubbing seems to be masked by the presence of other factors, as happens with atopy. "Having KC" (regardless of the grade) seems to be much more important than the degree of eye rubbing or the presence of Atopy in CT, and clearly other factors could be determinant in this relationship like the immune—inflammatory status.

In the multivariate analysis model, the KC history demonstrated a strong significative influence in CT of the studied population, and the second factor that seems to have more impact in CT is NLR index.

This study has some limitations which are worth noting. The main one has to do with the cross-sectional design of the research study, which clouds the determination of a causal relationship between the inflammatory markers, choroidal profile and KC development. Secondly, the limited sample size could have had some influence on the results, restricting the generalization of the data. It is reasonable to accept that with a larger sample some differences could be more pronounced and become significative. Another limitation, already described in previous reports, was the protocol used for choroidal profile evaluation. Using a single-line fovea-centered scan protocol, some alterations in the posterior pole choroidal profile may have gone unnoticed.

The inclusion of patients and controls with atopic disease (allergic asthma, atopic dermatitis and allergic rhinitis) seems not be a limitation, as the Atopy has a great relationship with KC development and the presence of atopy was carefully controlled in all analysis performed as part of the study. The hypothesis of high astigmatism distorting the images of the choroid was discarded given that retinal thickness was unaltered in the sample, and that the OCT choroidal profile obtained with and without scleral contact lenses in KC patients (eliminating corneal aberrations) were similar^40^.

In conclusion, this study reveals that serum inflammatory indices (NLR, SII and PLR) were significantly higher in KC patients, when compared to control subjects, and that these inflammatory biomarkers are associated with the increased CT presented by those patients. Even if nonspecific for the disease, these inflammatory indices are additional evidence of a role for systemic inflammation in the pathophysiology of KC and may help focus attention on the possibility of developing effective anti-inflammatory therapies for KC management.

## Methods

### Patients and data

We designed a cross-sectional, case–control study in which 40 patients with KC and 26 control subjects with no KC signs were enrolled.

All study participants were purposefully called in for inclusion in this study, and all examinations were performed by the same researcher (JPC) between January and March 2021.

Collected data included sex, age, patient’s ocular history, medical history (atopy, Eye rubbing, medications). Atopy was defined as the presence of at least one of the following conditions: allergic asthma, atopic dermatitis and allergic rhinitis. In all cases atopy history were carefully screened, based on allergology specialist consultation and laboratory test screening (prick tests and/or specific IgE for most common allergens—Phadiatop). Eye rubbing habits were evaluated and classified as: 0—none (never rub their eyes), 1—rarely (less than one time per week), 2—sometimes (between one and several times per week) or 3—frequently (everyday).

All patients performed a complete ophthalmic examination to exclude other ocular pathologies (best-corrected Snellen visual acuity (BCVA), biomicroscopic examination, intraocular pressure and fundus examination).

The morphological characterization of the cornea was performed using Pentacam HR (OCULUS Optikgeräte GmbH, Wetzlar, Germany). KC grade classification was based in Pentacam HR software topographical keratoconus classification (range 0–4). Tomographic parameters studied for comparative means were maximum keratometry (Kmax), minimum pachymetry (PachyMin) and Belin/Ambrósio deviation index (D-Index).

AL was evaluated with a IOL Master 500 (Carl Zeiss Meditec, Jena, Germany) and adjusted AL was calculated (AL—anterior chamber depth (ACD)) correcting the AL for the more pronounced ACD presented by KC patients.

The inclusion criteria were the following: patients aged between 18 and 30, with clinical and topographic KC signs and age-matched controls with a normal corneal topography. Exclusion criteria included: previous surgical intervention, other than corneal crosslinking more than 6 months before; history of corneal trauma or any other corneal pathology; existence of active systemic or ocular inflammation; current treatment with systemic or local anti-inflammatory drugs (only artificial tears were accepted in the month before the study inclusion); a history of hepatic, renal, metabolic, hematologic and immunologic diseases; any kind of infection in the month preceding the blood sample collection, since it might interfere with the results of blood samples and inflammatory molecules. When both eyes meet the inclusion criteria only right eyes were used for statistical analysis.

This study was approved by the local Ethics Committee (Project no. 190/18 CECHSJ/FMUP) and conducted in accordance with all the requirements of the Helsinki Declaration. Informed consent was obtained from each patient or control subject after full explanation of the procedures.

### Blood samples analysis

Venous blood samples were collected in the morning from the antecubital vein after a fasting period of 8–12 h. Blood counts, including lymphocyte, neutrophil, monocyte and platelet (all × 10^6^/mL), were performed by the method of laser-based flow cytometric impedance, using an automated blood cell counter (Sysmex XE-2100, Sysmex Corporation, Kobe, Japan). HDL-cholesterol values (mg/dL) were determined using conventional methods with a Beckman-Coulter AU5400 automated clinical chemistry analyzer (Beckman-Coulter).

The NLR was calculated by dividing the neutrophil count by the lymphocyte count, MHR was calculated by dividing the monocyte count by HDL-cholesterol level, PLR was calculated by dividing the platelet count by the lymphocyte count and SII was calculated as platelet count × (neutrophil/lymphocyte).

### Inflammatory molecules

The inflammatory biomarkers IL-1, IL-6 and TNF-alfa were analyzed in serum samples by a multiplexed immunoassay using a Human Cytokine/Growth Factor Magnetic Bead Panel Kit (Milliplex®Map Kit, Millipore, Cat.HCYTA-60K, EMD Millipore Corporation, Germany, 2019) and analyzed in the Luminex 200TM xMAPTM Technology (Luminex Corp., Austin, USA, 2009). The results were quantified based on the Median Fluorescence Intensity (MFI) data using a standard five parameter logistic (5-PL) curve fit created by the Luminex xPONENT ® Software (version 3.1).

### Choroidal imaging

The patients underwent enhanced depth imaging (EDI) spectral-domain optical coherence tomography (SD-OCT) using the Spectralis Heidelberg apparatus (Heidelberg Engineering Inc, Heidelberg, Germany). The choroidal imaging protocol was applied according to the previously published in 2019 by Pinheiro–Costa et al.^24^.

**Figure 6:**
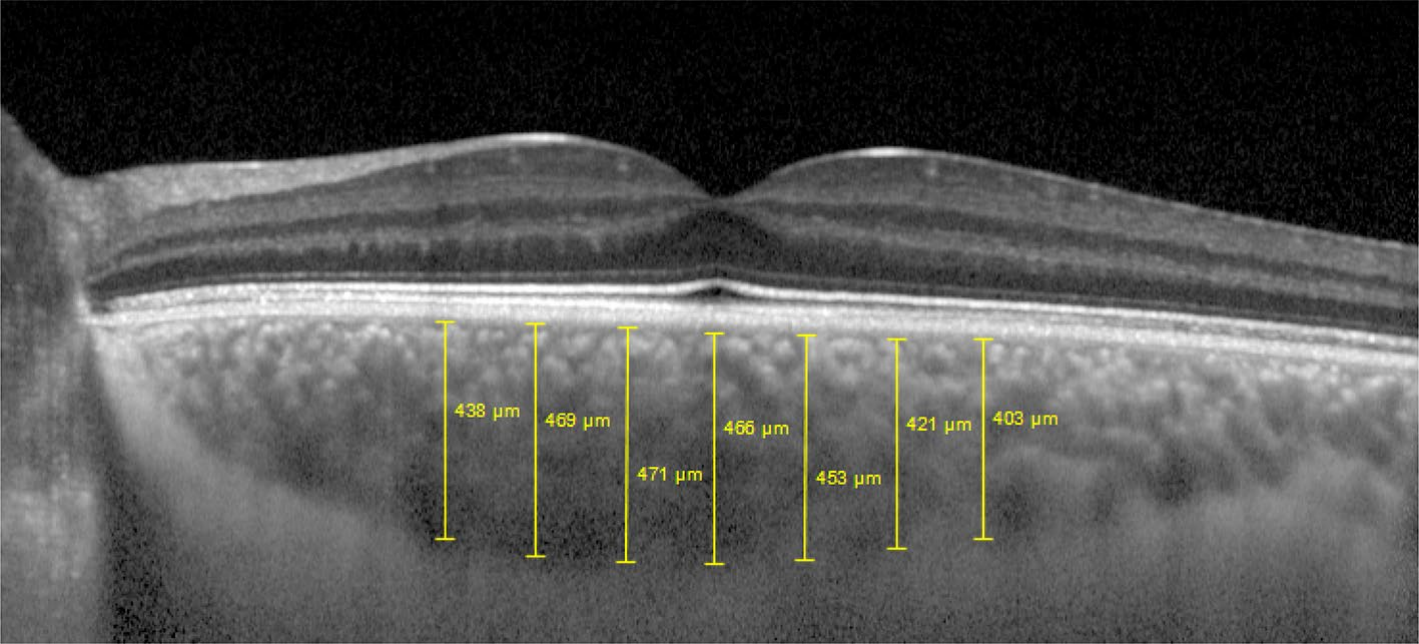
Representation of a CT measurement in a KC eye using the semiautomatic mode. Measurements were taken at the subfoveal choroid and at 500 µm intervals from the fovea, temporal 500 μm, 1000 μm, 1500 μm, and nasal 500 μm, 1000 μm and 1500 μm.

The SD-OCT scans were single 30-degree, B-scans centered on the fovea using the EDI function averaged 100 times. CT was measured on the horizontal OCT from the outer edge of the hyperreflective line—corresponding to the retinal pigment epithelium—to the choroidal-scleral junction, as illustrated in Fig. 6. Choroidal Thickness measurements were undertaken at subfoveal choroid (Subfoveal), nasal 500 μm (N500), 1000 μm (N1000) and 1500 μm (N1500) and temporal 500 μm (T500), 1000 μm (T1000), 1500 μm (T1500).

Only high-quality images were considered and all the images with a poor choroidal-scleral junction visibility were excluded. CT measurements were performed and confirmed manually by two masked independent observers (JPC and MLF).

### Statistical evaluation

The required sample for a survival test was calculated, using WinPepi® V11.65^41^, aiming for a statistical power of 80% and a one sided 0.05 significance level. Although bigger ratios disparities are described for NLR ratios or IL-6^17,19^, the sample was calculated at 40 (N1 = 20 and N2 = 20), with non-inferiority between groups. An expected error margin of 5% was assumed, so a sample of 42 at minimum was aimed.

Descriptive statistics are presented as total count and percentage for categorical variables like sex, eye rubbing habits and KC classification, and as mean (± standard deviation, SD) or median and range (25th and 75th percentiles) for continuous variables like CT and laboratory parameters. Determination of the normality of the distribution was achieved using the Kolmogorov–Smirnov test.

Chi-square or Fisher tests were used to compare proportions, while the Student's t or Mann–Whitney tests were used to compare the continuous variables between groups, depending on whether their distribution was normal or not.

Pearson's (normally distributed variables) or Spearman’s (variables with non-normal distribution) coefficients were used for bivariate correlations.

Multivariate linear regression analysis, using generalized linear models adjusted for age, AL, KC history, Atopy history, eye rubbing habits and inflammatory indices were performed to assess the influence of the various studied parameters in the CT. The independent influence of NLR and SII on Subfoveal CT was assessed by different models. These multivariate linear regression models were adjusted by atopic disease. Results were expressed as Beta estimate with the corresponding 95% confidence intervals (95% CI).

The significance level was set at 0.05. Statistical analysis was conducted using SPSS statistical software package version 26 (SPSS inc., Chicago IL., USA).

## Data Availability

The datasets generated during and/or analyzed during the current study are available from the corresponding author on reasonable request.
